# Publisher Correction: MLL-AF9 initiates transformation from fast-proliferating myeloid progenitors

**DOI:** 10.1038/s41467-020-14428-4

**Published:** 2020-01-29

**Authors:** Xinyue Chen, Daniel B. Burkhardt, Amaleah A. Hartman, Xiao Hu, Anna E. Eastman, Chao Sun, Xujun Wang, Mei Zhong, Smita Krishnaswamy, Shangqin Guo

**Affiliations:** 1 Department of Cell Biology, Yale University, New Haven, CT 06520 USA; 2 Yale Stem Cell Center, Yale University, New Haven, CT 06520 USA; 3 Department of Genetics, Yale University, New Haven, CT 06520 USA; 4 SJTU-Yale Joint Center for Biostatistics and Data Science, Shanghai Jiao Tong University, Shanghai, 200240 China

**Keywords:** Acute myeloid leukaemia, Oncogenes

Correction to: *Nature Communications* 10.1038/s41467-019-13666-5, published online 18 December 2019.

The original version of this Article incorrectly acknowledged Smita Krishnaswamy as a corresponding author. This has now been corrected in both the PDF and HTML versions of the Article.

