# Supplementary material for: MLL-AF9 initiates transformation from fast-proliferating myeloid progenitors
Source: Nat Commun. 2019 Dec 18;10:5767. doi: 10.1038/s41467-019-13666-5 (PMC6920141; doi:10.1038/s41467-019-13666-5)
Supplement: Supplementary file 1 — Supplementary Information [file 41467_2019_13666_MOESM1_ESM.pdf]

Supplementary Information

MLL-AF9 initiates transformation from fast-proliferating myeloid progenitors

Chen *et al*.

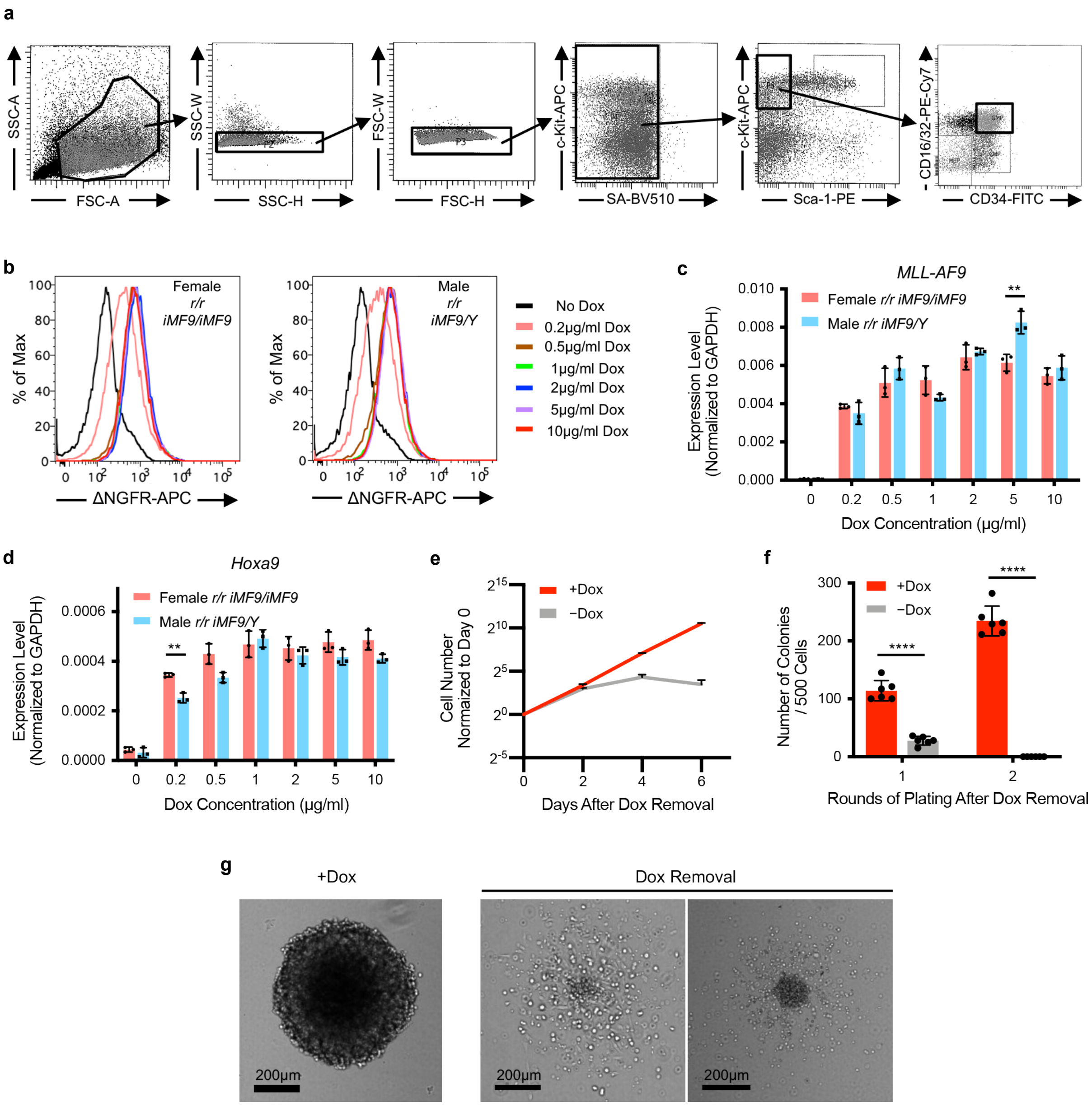

Supplementary Figure 1: Establishing an inducible MLL-AF9 knock-in mouse model.
a, Gating strategy to sort GMPs (Lin–c-Kit+Sca-1–CD34+CD16/32+). The initial cell population is bone marrow cells after lineage depletion.
b, FACS analyses of cell surface NGFR expression in male (*iMF9/Y*) and female (*iMF9/iMF9*) iMLL-AF9 GMPs treated with various concentrations of doxycycline (Dox) for 2 days. All mice were homozygous for *Rosa26:rtTA*, designated as *r/r*. All subsequent studies were performed using these same genotypes.
c, RT-QPCR analyses of *MLL-AF9* expression levels in male and female iMLL-AF9 GMPs treated with various concentrations of Dox for 2 days. n=3, p=0.0077 at 5μg/ml Dox concentration, for all the other pair-wise comparisons, p>0.1.
d, RT-QPCR analyses of *Hoxa9* expression levels in male and female iMLL-AF9 GMPs treated with various concentrations of Dox for 2 days. n=3, p=0.0017 at 0.2μg/ml Dox concentration, for all the other pair-wise comparisons, p>0.02.
e, Freshly-isolated iMLL-AF9 GMPs were cultured in 2μg/ml of Dox for 8 days, when the culture was split into +/–Dox conditions. Cell numbers were scored in each conditions at the indicated time (n=2).
f, Freshly-isolated iMLL-AF9 GMPs were plated in methylcellulose for two rounds in the presence of 2μg/ml of Dox, when the cells underwent another two rounds of replating in the presence of absence of Dox. The number of colonies in each of these replatings were scored. n=6 for each conditions, p<0.0001 for both rounds of replating.
g, Representative colony morphologies that grew in serially-replated GMPs, in the presence of Dox or after Dox removal. Scale bar: 200μm.
Results are presented as mean ± S.D.

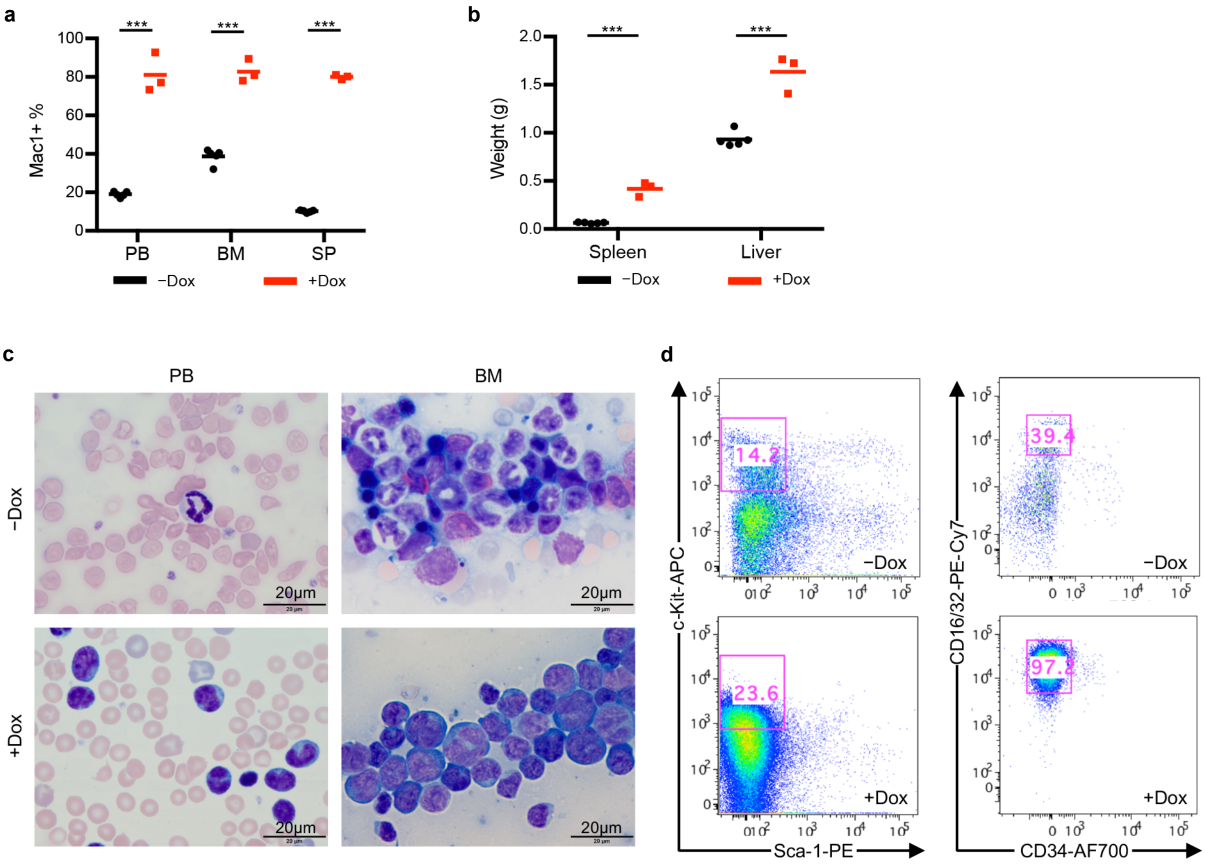

Supplementary Figure 2: iMLL-AF9 GMPs support AML development *in vivo*.

a, Myeloid cell percentage in peripheral blood (PB), bone marrow (BM), and spleen (SP) of recipient mice transplanted with iMLL-AF9 GMP. Samples were collected and analyzed 7 weeks post transplantation. p<0.001 for all comparisons. (n=5 for –Dox group, n=3 for +Dox group)

b, Spleen and liver weight of recipient mice transplanted with iMLL-AF9 GMPs. Samples were collected and analyzed 7 weeks post transplantation, p<0.001 for all comparisons. (n=5 for –Dox group, n=3 for +Dox group). Data from a representative cohort is shown.

c, Giemsa staining of representative peripheral blood (PB) smear and bone marrow (BM) smear from iMLL-AF9 GMP transplanted recipient mice 7 weeks post transplantation. Scale bar: 20µm.

d, FACS plots confirming the presence of L-GMPs in diseased mouse bone marrow.

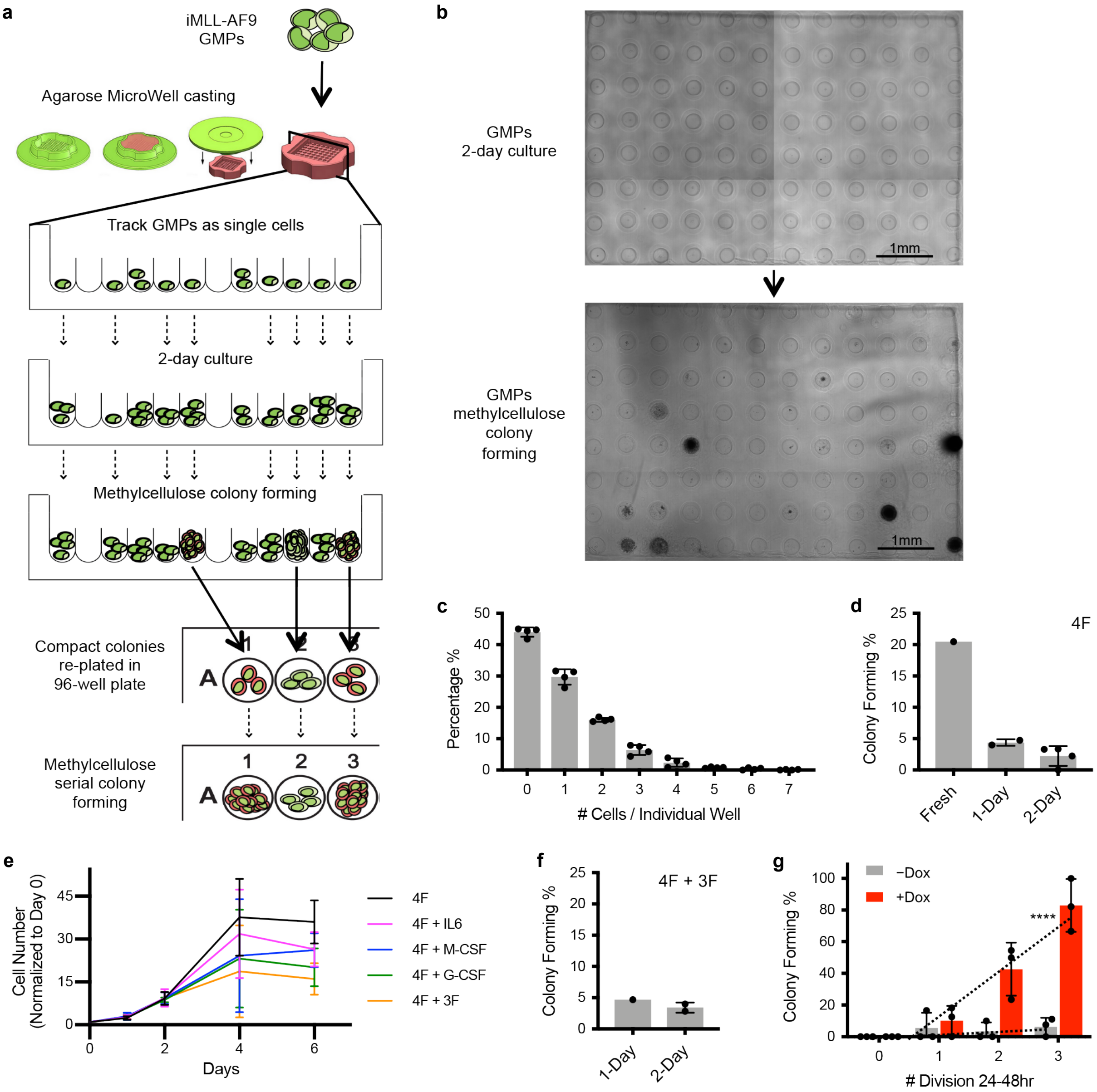

Supplementary Figure 3: Permissiveness for MLL-AF9 mediated transformation is associated with the intrinsic cycling rate of GMPs.

a, Detailed workflow to track transformation from single iMLL-AF9 GMPs, as shown also in Fig. 1d. A schematic cross-section view of the micro-wells is shown. Only the wells that contained single cells right after cell plating were continuously tracked. To confirm serial replating ability, colonies confined in each well were plucked and replated into single wells in a 96-well plate. The presence of compact colonies in 96-well plates was scored as successful replating events.

b, Representative images of one micro-well gel during liquid culture (top) and following methylcellulose colony formation (bottom). Cells shown in these images were GMPs transduced with a lentivirus expressing MLL-AF9, which shares the same coding sequence for the iMLL-AF9 allele. Images were taken with a 4X objective, and stitched to show the entire micro-wells. Scale bar: 1mm.

c, Proper cell plating conditions for obtaining large numbers of wells that contain single cells. Typical cell number distribution in individual wells when 75µl of cells resuspended at 2,500 cells/mL were loaded into one micro-well unit. Shown cell number distribution is a summary of four independent experiments, with 4-5 micro-well units analyzed for each experiment.

d, Primary colony forming efficiency in methylcellulose by single iMLL-AF9 GMPs, when they were freshly isolated or following one or two days of culture. During culture, GMPs were supplemented with IL3, mTPO, Flt3L, and mSCF (4F).

e, The proliferation rates of GMPs when cultured with additional cytokines. 4F stands for IL3, mTPO, Flt3L, and mSCF; 3F stands for IL6, M-CSF, and G-CSF.

f, Primary colony forming efficiency by single iMLL-AF9 GMPs, following one or two days of culture. During culture, GMPs were supplemented with IL6, M-CSF, and G-CSF, in addition to IL3, mTPO, Flt3L, and mSCF (4F+3F), in comparison to **d**.

g, Primary colony forming efficiency by iMLL-AF9 GMPs of different cycling rates, in the absence or presence of Dox. Cycling rates were defined by number of cell divisions during 24-48hr culture. Note this cycling rate was no longer the intrinsic cycle rate, as shown in Fig. 2d. Dotted lines represent linear regressions. n=3 for both +/–Dox conditions. For +Dox condition, y=28.13x-8.227, the slope is signifi-cantly non-zero, $p<0.0001$ as calculated by F-test; for –Dox condition, y=1.658x+1.302, p=0.3011.

Results are presented as mean±S.D.

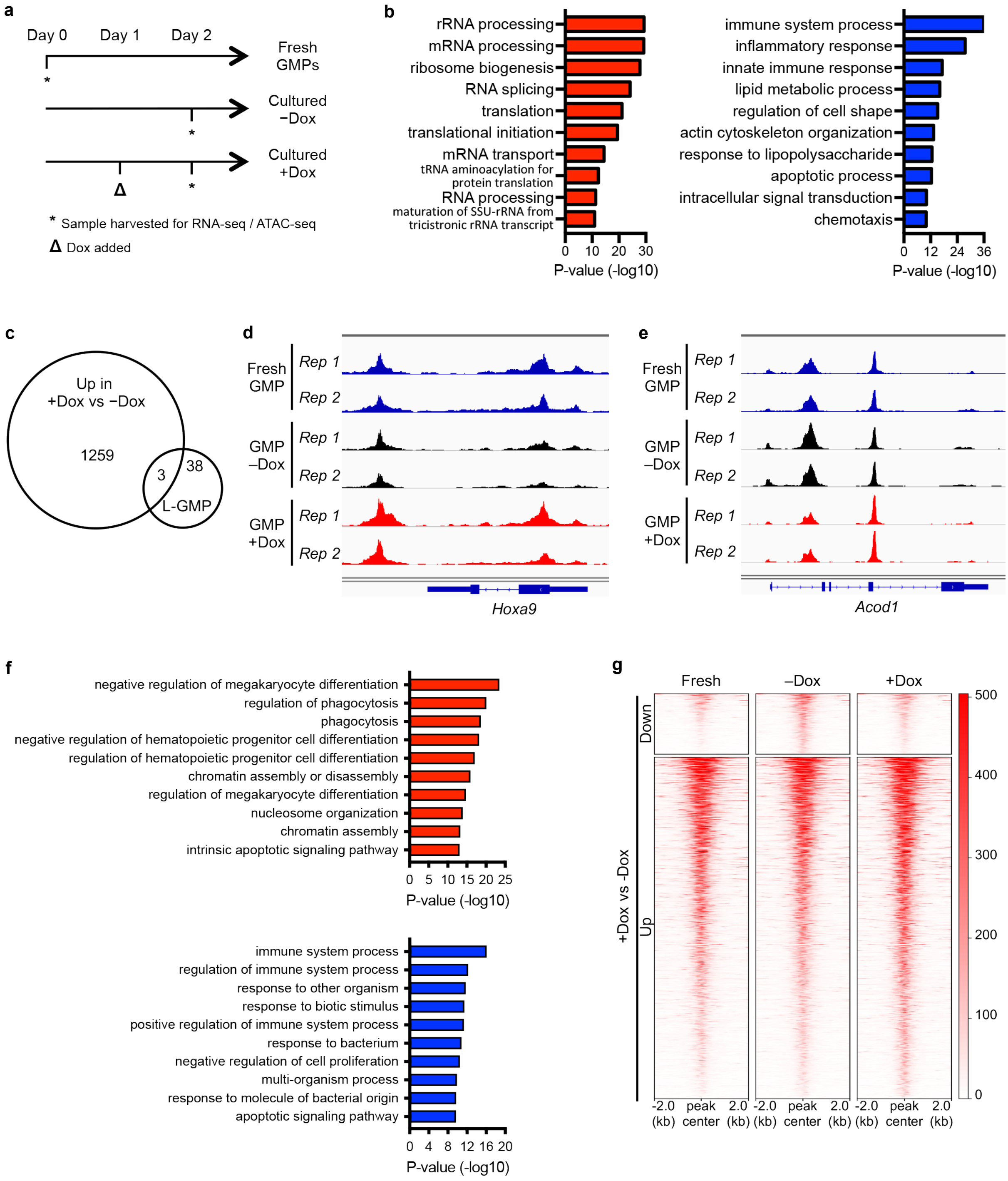

Supplementary Figure 4: MLL-AF9 helps to sustain the already-existing gene expression program in GMPs.
a, Schematic showing the timeline for iMLL-AF9 GMP treatment with Dox and sample harvest, for both RNA-Seq and ATAC-Seq analyses.
b, Gene ontology (GO) analysis for DEGs defined by comparing +Dox versus –Dox iMLL-AF9 GMPs. The top 10 up-regulated (red bars) and down-regulated (blue bars) biological process terms in +Dox GMPs are shown.
c, Venn diagram showing minimal overlap between the Dox up-regulated DEGs and the previously defined stem cell signature in L-GMPs.
d, Representative ATAC-seq tracks, with *Hoxa9* genomic regions as an example, showing increased chromatin accessibility in +Dox versus –Dox iMLL-AF9 GMPs.
e, Representative ATAC-seq tracks, with *Acod1* genomic regions as an example, showing decreased chromatin accessibility in +Dox versus –Dox iMLL-AF9 GMPs.
f, GO analysis of the differential ATAC-Seq peaks from iMLL-AF9 GMPs treated +/–Dox for 24 hours. The top 10 biological process terms up-regulated (red bars) and down-regulated (blue bars) in +Dox GMPs are shown.
g, Heatmap showing ATAC-seq peak intensities in iMLL-AF9 GMPs, fresh or cultured +/–Dox for 24 hours. Regions Down (697) or Up (3929) were defined by comparing +Dox GMPs with –Dox GMPs.

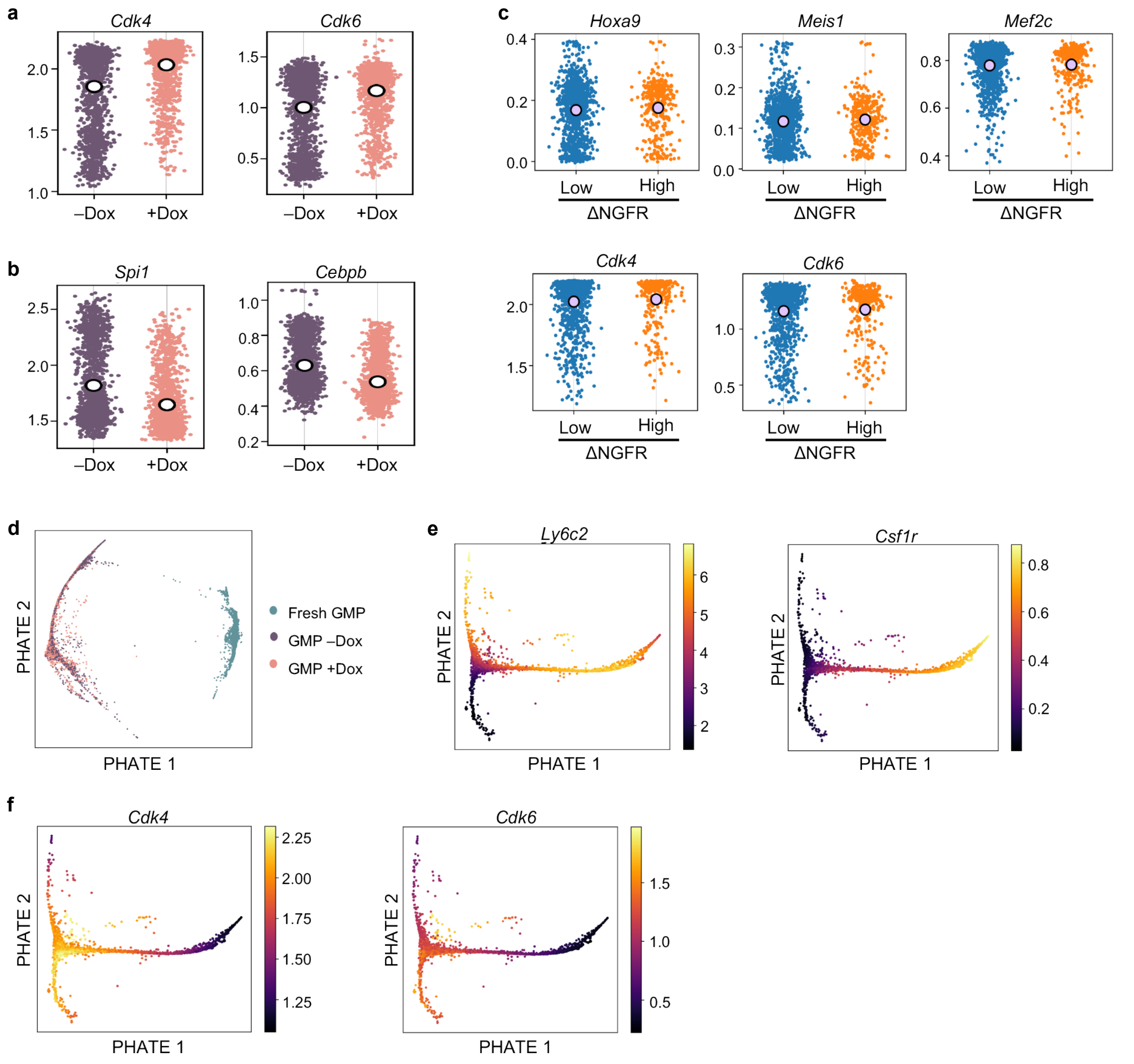

Supplementary Figure 5: Preservation of the heterogeneous gene expression states following iMLL-AF9 induction as analyzed by scRNA-Seq.

a, Swarm plots showing the expression levels of *Cdk4* and *Cdk6* in cultured GMPs in the presence or absence of Dox. White dots indicate mean of each sample.

b, Swarm plots showing the expression levels of *Spi1* and *Cebpb* in cultured GMPs in the presence or absence of Dox. White dots indicate mean of each sample.

c, Expression levels of *Hoxa9*, *Meis1*, *Mef2c*, *Cdk4*, and *Cdk6* in +Dox GMPs. Cells were separated into two groups based on the co-expressed *ΔNGFR* transgene: Low and High. Pink dots indicate mean of each sample.

d, PHATE plot displaying all three GMP samples, cultured GMPs +/–Dox and freshly-isolated GMPs.

e, Pseudo-color denoting the expression levels of *Ly6c2* or *Csf1r*, overlaid onto the PHATE plot for the cultured GMPs including both +Dox and –Dox cells.

f, Pseudo-color denoting the expression levels of *Cdk4* or *Cdk6*, overlaid onto the PHATE plot for the cultured GMPs including both +Dox and –Dox cells.

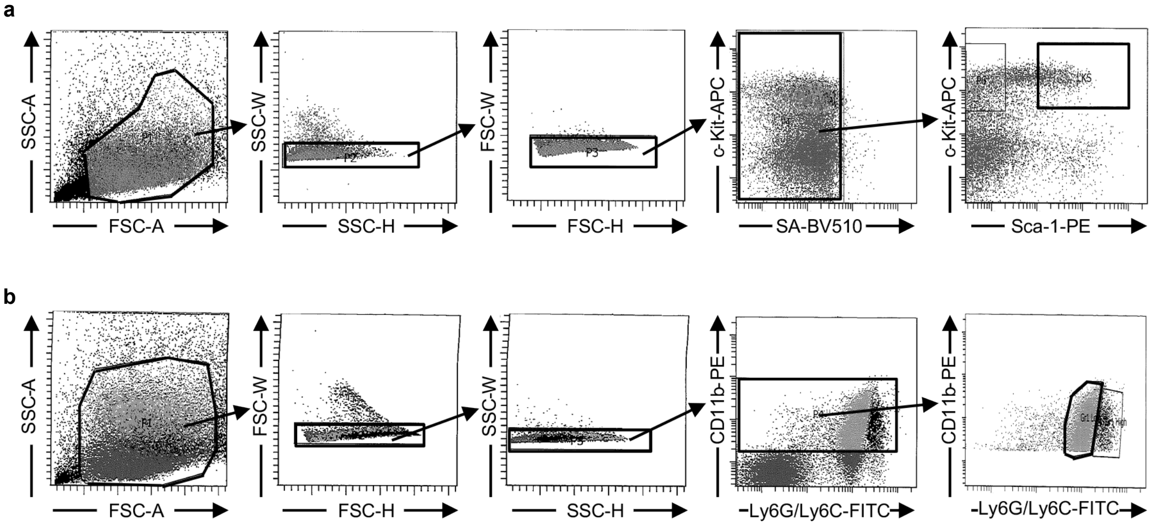

Supplementary Figure 6: Gating strategies for LKS and Mac1+ cells.
a, Hematopoietic stem and progenitor cells upstream of GMP were sorted as LKS (Lin–cKit+Sca-1+). The initial cell population is bone marrow cells after lineage depletion.
b, Differentiated myeloid cells (Mac1+) were sorted as Mac1+Gr1^{int}. The initial cell population is bone marrow Lin+ cells recovered from LD Columns.

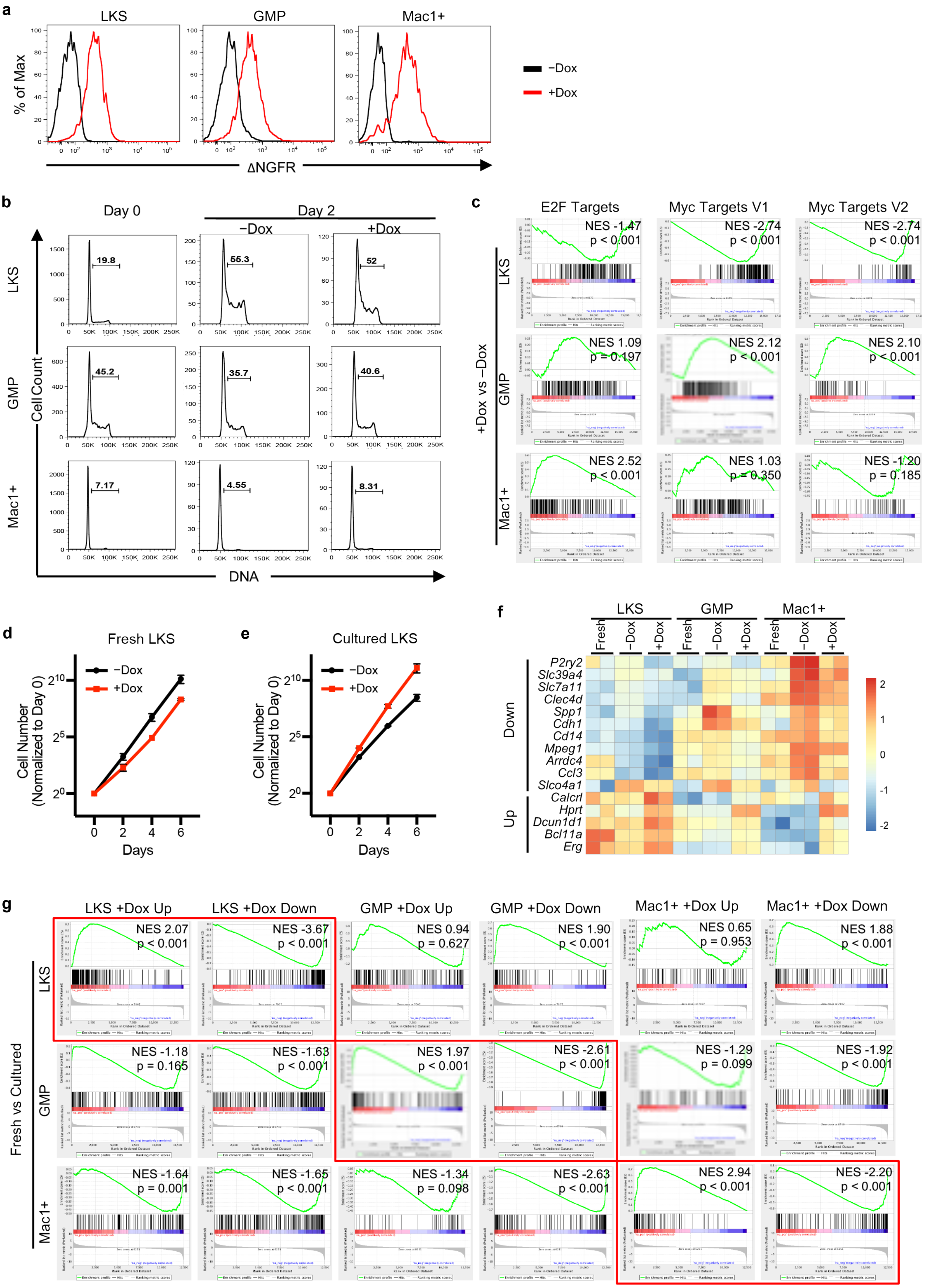

Supplementary Figure 7: The primary gene expression changes to MLL-AF9 induction is cell-context dependent.
a, FACS plots of hNGFR staining in LKS, GMP and Mac1+ cells, confirming transgene induction in all cell types.
b, FACS plots of Hoechst staining of DNA content in specified fresh cell types, or after 2 days of culture +/–Dox. Gates denote the frequency of cells in S/G2/M phases of the cell cycle.
c, Distinct proliferative responses to MLL-AF9 induction in LKS, GMP and Mac1+ cells, as determined by Gene Set Enrichment Analysis (GSEA). Note that only GMPs displayed consistent positive enrichment for all cell cycle gene sets.
d, Proliferation response to MLL-AF9 induction in fresh LKS cells (n=2). Dox was added to fresh LKS at Day 0.
e, Proliferation response to MLL-AF9 induction in cultured LKS cells (n=2). Dox was added after 4 days. x-axis denotes the time (Days) after Dox addition. Note the switched position of the black line and red line as compared to those in **d**.
f, Heatmap showing expression levels of 11 commonly down-regulated and 5 commonly up-regulated genes in all three cell types. Log2TPM of each gene was row normalized.
g, The Dox-induced DEGs, similar to those defined in Fig. 5c but at p<0.01, were queried against the gene expression changes occurred during culture across all three cell types by GSEA. For GMPs, only the top 200 DEGs were used. Note the DEGs were only enriched for their own respective cell type, but not across the other two cell types. DEGs against their respective cell types were highlighted by red box, and are the same as shown in Fig. 5e.

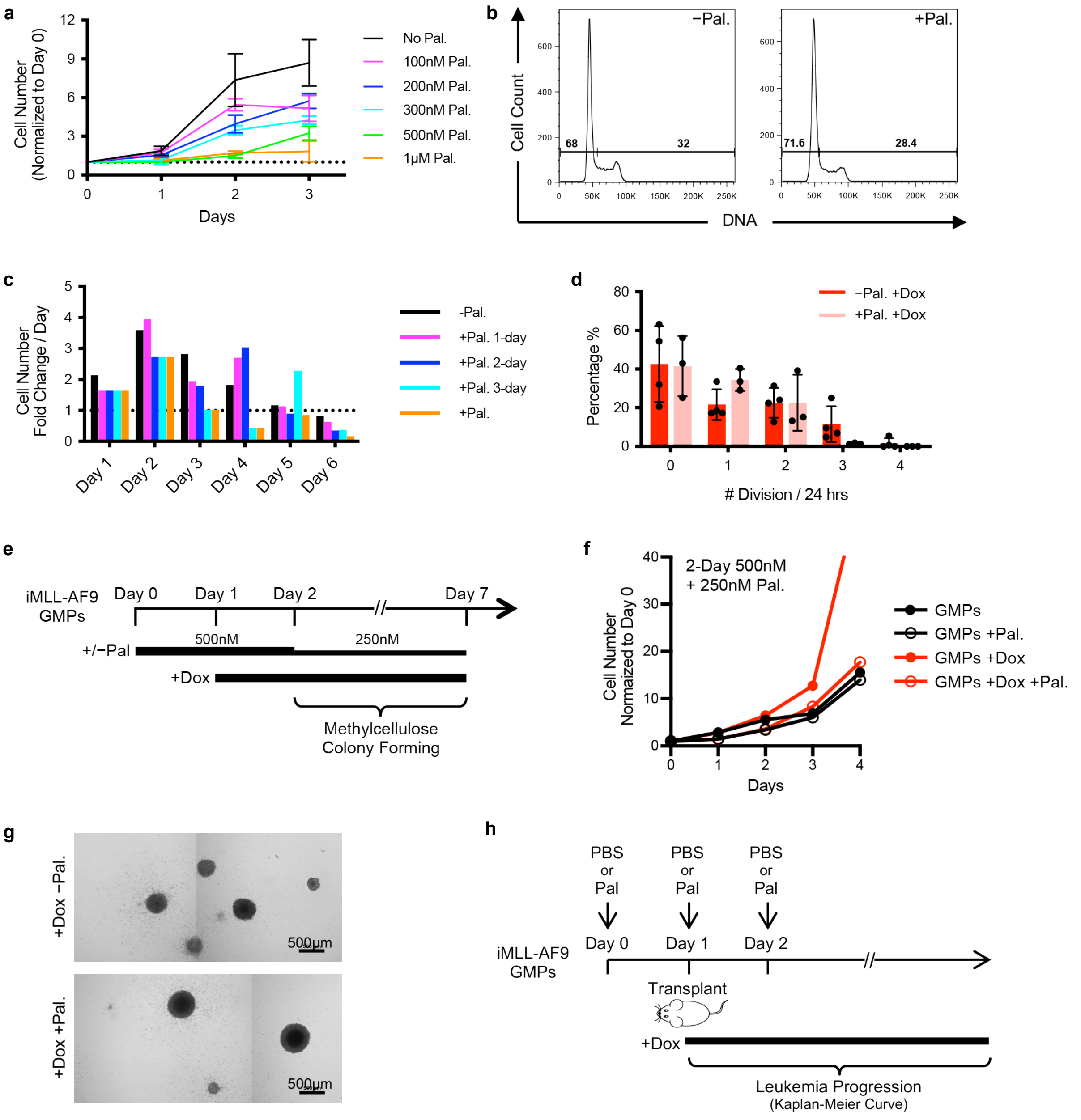

Supplementary Figure 8: Modification of the initial cell state by mild cell cycle inhibition mitigates MLL-AF9 mediated transformation.

a, GMP proliferation rates in the presence of different concentration of palbociclib. Proliferation was determined by scoring the number of viable cells in each condition at daily intervals. Note the minimal reduction in cell numbers at 500 nM (green line).

b, FACS plots of Hoechst staining of DNA content of GMPs following a 2-day culture in the presence of 500nM palbocilib. Gates denote the frequency of cells in S/G2/M phases of the cell cycle.

c, The proliferation rates of GMPs treated with 500nM palbociclib for various durations. For all treated conditions, palbociclib was added on Day 0, and washed out following the indicated duration. Cell proliferation rate (y-axis) was measured as cell number normalized to that of the same condition on the previous day. Dotted line (y=1) indicated no change in cell number during the indicated two consecutive days. Note the recovery of proliferation in all treated conditions following palbociclib removal, except for when it was present throughout.

d, Cell cycle rate distribution of iMLL-AF9 GMPs cultured in micro-wells in the presence or absence of palbociclib (+/–Pal.). Palbociclib was added at 0hr. Dox was added at 24hr, and cell cycle rate was determined from 24hr to 48hr. Error bars represent standard deviation. Data is pooled from 4 (–Pal.) and 3 (+Pal.) independent experiments. Detailed measurements are shown in Extended Data Table 2.

e, Schematics illustrating the timeline for colony formation by bulked cultured iMLL-AF9 GMPs +/–Pal..

f, The proliferation rates of bulk cultured iMLL-AF9 GMPs in various treatment conditions following the first 4 days of the schematics shown in **e**.

g, Representative images showing colony morphologies from bulked cultured iMLL-AF9 GMPs +/–Pal.. Scale bar:500μm.

h, Schematics illustrating the timeline for *in vivo* leukemogenesis by iMLL-AF9 GMPs, following the mild and temporary treatment by palbociclib. PBS was the vehicle control for palbociclib.

Results are presented as mean ± S.D.

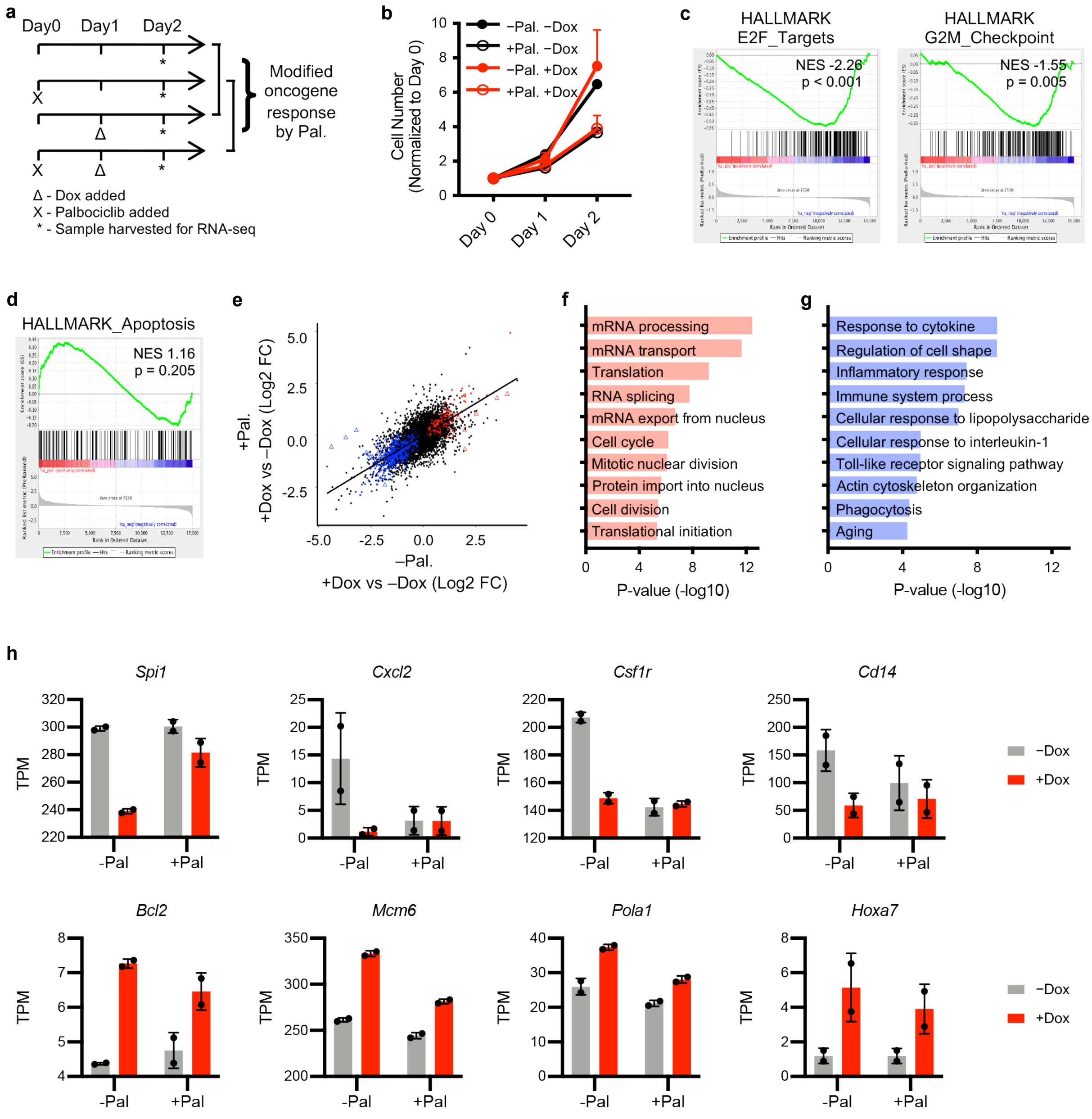

Supplementary Figure 9: Modification of the initial cell state by cell cycle inhibition mitigates MLL-AF9 mediated changes in gene expression.

a, Timeline schematics illustrating treatment with a CDK4/6 inhibitor palbociclib or vehicle control (+/−Pal.) for iMLL-AF9 GMPs, prior to their culture, in the presence or absence of Dox (+/−Dox). All cells were harvested for RNA-seq analyses after a total of 2 days culture. Comparisons used for analyses were indicated by the brackets on the right. Fresh GMPs were also included in the analysis.

b, The proliferation rates of iMLL-AF9 GMP samples used for RNA-seq (n=2).

c, GMPs treated with 500nM palbociclib for 2 days down-regulated cell cycle pathways as determined by GSEA.

d, GMPs treated with 500nM palbociclib for 2 days did not activate apoptotic pathways significantly as determined by GSEA.

e, The modified gene expression response to MLL-AF9 induction (+/–Dox) by palbociclib (+/–Pal.). Individual genes were plotted as single dots or triangles. Black line is the linear regression of the scatter plot, showing positive correlation. Significantly up-regulated and down-regulated genes are marked in red and blue, respectively. Genes similarly changed by Dox irrespective of the presence of Pal. are denoted as dots, while those changed only in the absence of palbociclib are denoted as triangles.

f, GO analysis for Dox up-regulated DEGs only in the absence of palbociclib (497 genes as shown in Fig. 6i). The top 10 biological process terms are shown.

g, GO analysis for Dox down-regulated DEGs only in the absence of palbociclib (692 genes as shown in Fig. 6i). The Top 10 biological process terms are shown.

h, Representative gene expressions in iMLL-AF9 GMPs treated with Pal., in the absence and presence of Dox.

Results are presented as mean±S.D.

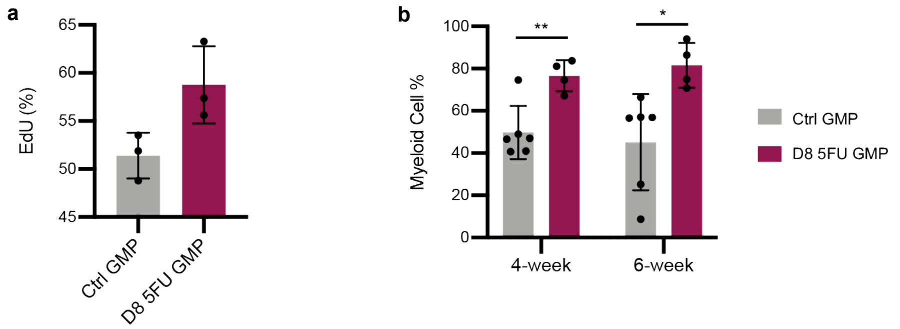

Supplementary Figure 10: Activated GMPs during emergency myelopoiesis support more efficient transformation.
a, Freshly-isolated GMPs from either untreated animals or those at 8 days after 5FU injection were stained and analyzed for EdU+ cells, following a 1 hour pulse label. n=3, p=0.0527 as calculated by unpaired t-test.
b, Myeloid cell (Mac1+) percentages in peripheral blood of recipient mice at 4- or 6-weeks after transplantations. n=6 for Ctrl GMP, n=4 for D8 5FU GMP, p=0.0052 for 4-week, p=0.0184 for 6-week, as calculated by unpaired t-test.
Results are presented as mean±S.D.

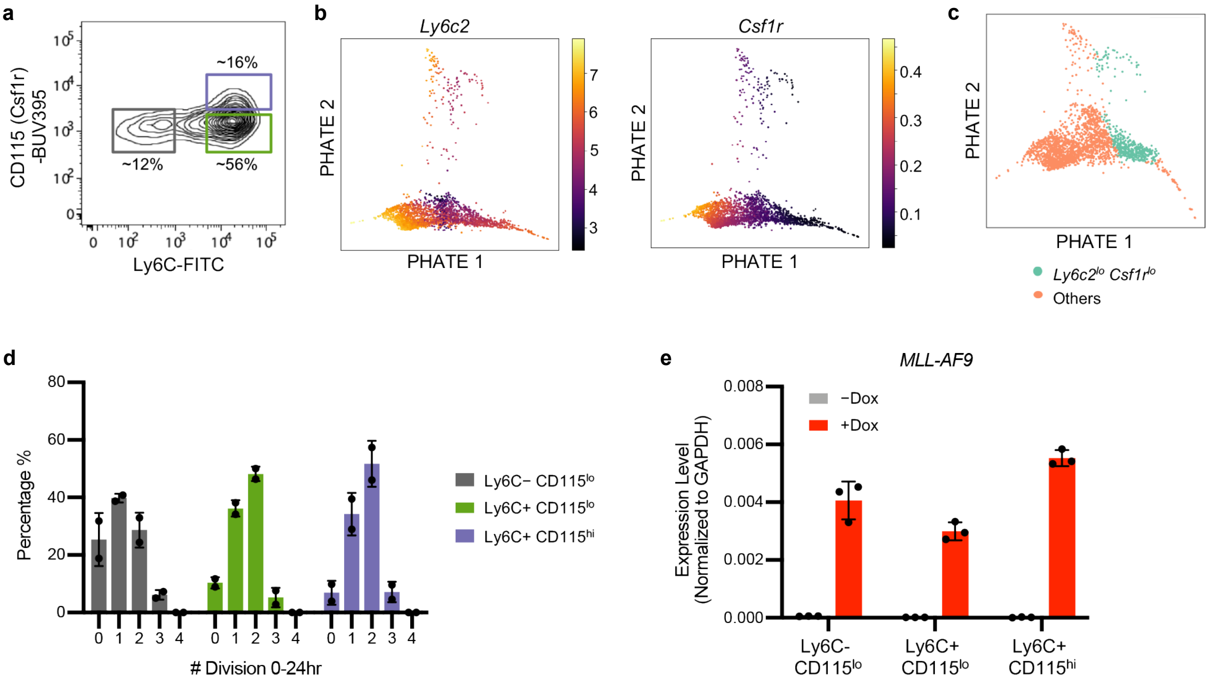

Supplementary Figure 11: Ly6C–CD115lo GMPs are further enriched for MLL-AF9 mediated transformation.

a, Representative FACS plot showing three subsets of GMPs based on surface markers, Ly6C and CD115. Population frequencies are indicated next to corresponding gates. The starting cell population is GMP as gated according to Supplementary Figure 1a.

b, PHATE plot of single cell RNA-seq data on fresh GMPs. Pseudo-color denotes expression levels of *Ly6c2* or *Csf1r*. **c,** PHATE plot showing the *Ly6c2*lo*Csf1r*lo GMP subset in fresh GMPs.

d, Cell cycle rate distribution of each GMP subpopulations during 0-24hr of culture (n=2). iMLL-AF9 GMPs were analyzed with Dox added at 0hr. Note the similar cell cycle distributions as shown in Fig. 8a, where Dox was not added.

e, RT-QPCR showing MLL-AF9 transgene expression levels in the three subsets of GMPs, following 2 days of culture +/– Dox (n=3).

Results are presented as mean±S.D.

Supplementary Table 1. Single-cell cell cycle measurements (+/−Dox)

−Dox	Total cells	# Division 0-24hr				
		0	1	2	3	4
Rep. 1	75	15 (20%)	44 (58.7%)	13 (17.3%)	3 (4%)	-
Rep. 2	60	20 (33.3%)	26 (43.3%)	13 (21.7%)	1 (1.7%)	-
Rep. 3	133	37 (27.8%)	42 (31.6%)	51 (38.3%)	3 (2.3%)	-
**+Dox**	**Total cells**	**# Division 0-24hr**				
		0	1	2	3	4
Rep. 1	83	28 (33.7%)	40 (48.2%)	15 (18.1%)	-	-
Rep. 2	148	36 (24.3%)	69 (46.6%)	32 (21.6%)	11 (7.4%)	-
Rep. 3	147	31 (21.1%)	64 (43.5%)	50 (30.4%)	2 (1.4%)	-
**−Dox**	**Total cells**	**# Division 24-48hr**				
		0	1	2	3	4
Rep. 1	75	47 (62.7%)	15 (20%)	11 (14.7%)	2 (2.7%)	-
Rep. 2	60	38 (63.3%)	6 (10%)	7 (11.7%)	9 (15%)	-
Rep. 3	133	59 (44.4%)	40 (30.1%)	20 (15.0%)	13 (9.8%)	1 (0.8%)
**+Dox**	**Total cells**	**# Division 24-48hr**				
		0	1	2	3	4
Rep. 1	83	47 (56.6%)	7 (8.4%)	22 (26.5%)	7 (8.4%)	-
Rep. 2	148	60 (40.5%)	8 (5.4%)	52 (35.1%)	28 (18.9%)	-
Rep. 3	147	53 (36.1%)	22 (15.0%)	51 (34.7%)	21 (14.3%)	-

Supplementary Table 2. Single-cell cell cycle measurements (+/−Pal.)

**−Pal.**	**Total cells**	**# Division / 24hr**				
		0	1	2	3	4
Rep. 1	87	55 (63.2%)	15 (17.2%)	11 (12.6%)	6 (6.9%)	-
Rep. 2	137	44 (32.1%)	46 (33.6%)	33 (24.1%)	13 (9.5%)	1 (0.7%)
Rep. 3	179	37 (20.7%)	31 (17.3%)	56 (31.3%)	45 (25.1%)	10 (5.6%)
Rep. 4	125	68 (54.4%)	23 (18.4%)	28 (22.4%)	6 (4.8%)	-
**+Pal.**	**Total cells**	**# Division / 24hr**				
		0	1	2	3	4
Rep. 1	151	85 (56.3%)	44 (29.1%)	20 (13.2%)	2 (1.3%)	-
Rep. 2	158	40 (25.3%)	53 (33.6%)	62 (39.2%)	3 (1.9%)	-
Rep. 3	79	34 (43.0%)	32 (40.5%)	12 (15.2%)	1 (1.3%)	-

Supplementary Table 3. Oligo sequences

**Real-time PCR primers**	
Gapdh	GGTGCTGAGTATGTCGTGGAG
	GGCGGAGATGATGACCCTTT
MLL-AF9	GAATGCAGGCACTTTGAACA
	TGCCTTGTCACATTCACCAT
HoxA9	TAAACCTGAACCGCTCTCGG
	CCGCTCTCATTCTCGGCATT
Meis1	CGCCAGGGCTGCAAAGTAT
	AGGGTGTCCAGGAATGTACG
Cdk4	ATGGCTGCCACTCGATATGAA
	TCCTCCATTAGGAACTCTCACAC
Cdk6	GGCGTACCCACAGAAACCATA
	AGGTAAGGGCCATCTGAAAACT
**Genotyping PCR primers**	
MLL-AF9 KI	CTAGATCTCGAAGGATCTGGAG
	ATACTTTCTCGGCAGGAGCA
MLL-AF9 WT	GTCATAGGAACTGCGGTCGT
	GCTGGGATTTGAACTCAGGA
rtTA	AAAGTCGCTCTGAGTTGTTAT
	GCGAAGAGTTTGTCCTCAACC
	GGAGCGGGAGAAATGGATATG

Supplementary Table 4. Flow cytometry antibodies

Antibody	Clone	Vendor
CD271 (NGFR) - APC	ME20.4	BioLegend®
Ly-6G/6C - Biotin	RB6-8C5	BD Pharmingen™
CD3e - Biotin	145-2C11	BD Pharmingen™
CD45R/B220 - Biotin	RA3-6B2	BD Pharmingen™
CD11b - Biotin	M1/70	BD Pharmingen™
Ter119 - Biotin	Cat. 553672	BD Pharmingen™
CD8a - Biotin	53-67	BD Pharmingen™
CD4 - Biotin	GK1.5	BD Pharmingen™
CD117 - APC	2B8	BD Pharmingen™
Ly-6A/E (Sca-1) - PE	D7	eBioscience
Streptavidin - BV510	Cat. 563261	BD Horizon™
CD16/32 - PE-Cy7	93	eBioscience
CD34 - Alexa Fluor® 700	RAM34	BD Pharmingen™
CD34 - FITC	RAM34	BD Pharmingen™
CD11b (Mac1) - APC	M1/70	eBioscience
CD11b (Mac1) - PE	M1/70	BD Pharmingen™
Ly6G/Ly6C (Gr1) - FITC	RB6-8C5	BD Pharmingen™
CD45.1 - BV711	A20	BD Horizon™
CD45.2 - Pacific Blue™	104	BioLegend®
Ly6C - FITC	AL-21	BD Pharmingen™
CD115 (CSF-1R) - BUV395	T38-320	BD OptiBuild™
